# Materia Medica and Therapeutics: An Introduction to the Rational Treatment of Disease

**Published:** 1884-09

**Authors:** 


*Materia Medica and Therapeutics: an Introduction to the Rational Treatment of Disease.* By J. MITCHELL BRUCE, M.A. Aberd., M.D. Lond. London: CASSELL & Co., Limited. 1884.

IN the series of Cassell's *Students' Manuals*, the volume on *Materia Medica and Therapeutics*, by Dr. Mitchell Bruce, has been forwarded to us, and fully maintains the standard of excellence displayed by its predecessors. The size and price of the book were, we presume, restricted within somewhat narrow limits; wherefore it happens that certain departments of the subject are treated in rather a meagre way, compared to other departments. A book designed for students, in order to be completely useful, must be sufficiently comprehensive in all sections of a subject to enable them to pass their examinations in that subject; and as, under the present arrangements of the Examining Boards, students are examined in Materia Medica and Therapeutics at a comparatively early stage of their education, they are naturally supposed to have a more extended acquaintance with Materia Medica and Pharmaceutical Chemistry than with Therapeutics. In order to pass his examinations, the student must resort to some other book from which to learn sufficient Pharmaceutical Chemistry, as the subject is but very cursorily treated by Dr. M. Bruce.

This defect, however, being set aside—and it is one which it is most easy for the student to remedy—we have nothing but unqualified praise to assign to the book. There have been several books on Materia Medica published in the English language recently, and at least one more important work is advertised to appear shortly in an English form. Dr. Bruce has given us a veritable *multum in parvo*, which surpasses in matter and manner anything which we have had hitherto in the form of a Students' Manual. The method is truly scientific; the chaos of heterogeneous empiricism in which the Therapeutics of yesterday were expressed is replaced by precise statement of definite facts, to form the Therapeutics of to-day. The actions of remedies are given in their physiological and therapeutical relations, though, perhaps, not quite so distinctly as is done in some other hand-books of Therapeutics; and while every familiar drug is fully treated of, novelties, as chinolin, kairin, cascara sagrada, convallaria, and such like, are also well described. The sections on General Therapeutics are a valuable innovation, and will prove of much service to the advanced student, if he is capable not only of reading, but also of inwardly digesting them.

The not far distant issue of a new edition of the *British Pharmacopœia* will render necessary a revision of the text-books of Materia Medica now in use. We shall hope to see a second edition of Dr. Bruce's manual continue then, as it does now, to surpass its competitors *longo intervallo*.

